# Measuring Oscillating Walking Paths with a LIDAR

**DOI:** 10.3390/s110505071

**Published:** 2011-05-06

**Authors:** Mercè Teixidó, Tomàs Pallejà, Marcel Tresanchez, Miquel Nogués, Jordi Palacín

**Affiliations:** Department of Computer Science and Industrial Engineering, University of Lleida, Jaume II, 69, 25001 Lleida, Spain; E-Mails: mteixido@diei.udl.cat (M.T.); tpalleja@diei.udl.cat (T.P.); mtresanchez@diei.udl.cat (M.T.); mnogues@diei.udl.cat (M.N.)

**Keywords:** gait trajectory, gait measurement, laser range sensor, LIDAR, oscillatory path, alcohol intake

## Abstract

This work describes the analysis of different walking paths registered using a Light Detection And Ranging (LIDAR) laser range sensor in order to measure oscillating trajectories during unsupervised walking. The estimate of the gait and trajectory parameters were obtained with a terrestrial LIDAR placed 100 mm above the ground with the scanning plane parallel to the floor to measure the trajectory of the legs without attaching any markers or modifying the floor. Three different large walking experiments were performed to test the proposed measurement system with straight and oscillating trajectories. The main advantages of the proposed system are the possibility to measure several steps and obtain average gait parameters and the minimum infrastructure required. This measurement system enables the development of new ambulatory applications based on the analysis of the gait and the trajectory during a walk.

## 1. Introduction

Human movement has become an interesting research topic for understanding how the human body works [1–3]. The measurement of the walking and gait patterns has many applications. It can be used for specific applications such as identification [4,5], gender classification [6], crime scene reconstruction [7], *etc.* The detection of anomalies in the walking path and in gait patterns can be used as an early indicator of some illnesses related with diabetes [8], osteoarthritis [9], cerebral paralysis, such as Parkinson’s disease [10–13], or as a tool to evaluate the likelihood of falls among the elderly [14,15].

In [16], a general view of some gait analysis techniques were presented and compared to evidence their positive and negative aspects. The techniques were divided into different groups, depending on the technology used in the assessment of footfall timing (computation of both times and displacements of the foot during contact time) such as goniometry, cameras, systems of video cameras, other motion analysis systems, force plates and using accelerometers. Technically, the parameters of the gait can be obtained using several methodologies: attaching external markers to the human body [4,5,17,18], attaching accelerometers and gyroscopes to the body [19,20], attaching electrogoniometers [21–23], embedding sensors in shoes [24], analyzing floor pressure profiles [25,26], identifying the silhouette of the walker [27], *etc.* The information obtained with the sensors can be integrated in an articulated pendulum-like motion model [28] to get an estimate of the body movement. However, the estimate of the walking trajectory requires a large and expensive measurement facility [29].

Some efforts have been made to detect walk anomalies through the analysis of gait parameters. In [30], men’s displacements were registered with a photographic method and analyzed in order to provide a pattern of pelvic and thoracic rotation and thus detect abnormal gait. They concluded that the deviation of the selected parameters in a normal gait were too high to detect anomalies. In [8], 15 subjects were examined to determine the effects of diabetes on gait parameters. They concluded that diabetes indeed affects the gait. In [9], the effect of a walking rehabilitation program in patients with osteoarthritis of the knee was evaluated by analyzing gait parameters. In [12,13], the stride length-cadence abnormalities caused by Parkinson’s disease were studied. In [14], changes in the kinematics of elderly people were studied. They concluded that people that used to fall showed less stable gait patterns while walking.

The ingestion of alcohol is another source of anomalies in the gait. In [31], the gait patterns generated in supervised walking (with a specific cadence) before and after alcohol ingestion were observed. The authors concluded that even young adults showed a decline in static balance ability, step stride, gait velocity and cadence with moderate alcohol ingestion. In [32], the effect of alcohol on walk stability was studied with the conclusion that a blood alcohol concentration of less than 0.4 mg/mL induces instability in the gait and the stride length increases as this concentration increases. In [28] and [33], the sway during the walk before and after alcohol ingestion was studied under the Romberg-test conditions with eyes open and closed. They observed that the sway area was longer after drinking alcohol in both situations.

### 1.2. Contributions of This Work

The measurement of oscillating or arbitrary walking trajectories can mainly be performed using complex vision based systems [18,29]. Alternative systems such as based in pressure carpets have measurement areas with a limited width and provides information only when the foot is in contact with the floor [26]. Other systems based on the use of attached sensors provide information of the articulations but not on the walking path [19,20].

The main contribution of this work is the proposal of using a small size terrestrial LIDAR to measure oscillating walking trajectories in a large experimentation area. Two specific trajectory parameters are proposed to evaluate the oscillation of the walking path relative to an ideal straight path. Two experiments are proposed to validate the measurement system and obtain specific information about selected gait parameters in a straight and oscillating walk. An additional third experiment is also proposed to get specific information of the anomalies in the gait and in the walking path under the effect of moderate alcohol ingestion.

The detection of anomalies in the gait and in the walking path has many applications in medicine as the first effects of some illnesses affect the motor system and then the gait. The use of a portable terrestrial LIDAR enables the development of new measurement systems ready for ambulatory use. Another potential application relays on the correlation of some trajectory parameters with the ingestion of alcohol [34]. In this case, the use of a LIDAR as a main (or discriminative) sensor may represent a substantial increment in the number of subjects measured per hour relative to current measurement tools [35].

### 1.3. Related Work

The use of LIDAR laser range sensors for human locomotion measurement without external markers has been proposed recently by many authors [36–39] but this technology is still under development and has not been included in some recent reviews [16]. Currently, LIDAR sensors are used mainly to detect obstacles and identify people walking. In [37] a system with a laser scanner to improve pedestrian classification using target appearance was proposed. The state and the true contours of each target are recursively estimated and can then be used for accurate classification. In [38], a mobile robot using a LIDAR uses a sensor data segmentation module and a feature detection to estimate a curvature-based environment description with the aim of extracting features of dynamic environments and detecting if a moving object can be classified as a pedestrian. The specific analysis of gait parameters using a LIDAR was proposed in [39], where a complete measurement procedure was defined and the advantages and disadvantages enumerated. The main disadvantage stated was that only planar information of the position of the legs at a fixed height was obtained, although multiple laser range sensors at different heights could be used to obtain full body motion parameters [36].

## 2. Material and Methods

### 2.1. Subjects

The subjects collaborating in this work were all volunteers in an age range between 21 and 32 years and an average and standard deviation of 25 and 4 years respectively, and without any impairment. The purpose and procedure of each experiment were explained in detail to the volunteers before starting any measurement. Two volunteers that occasionally drank soft alcoholic beverages (one man and one woman) were selected for a specific experiment designed to evaluate the effects of moderate alcohol ingestion on their gait parameters. The measurement procedure related to the alcohol intake was developed with the collaboration and supervision of the Lleida section of the Police Force of the Government of Catalonia (Mossos d’Esquadra).

### 2.2. Apparatus

#### 2.2.1. Terrestrial LIDAR

**Figure 1.**
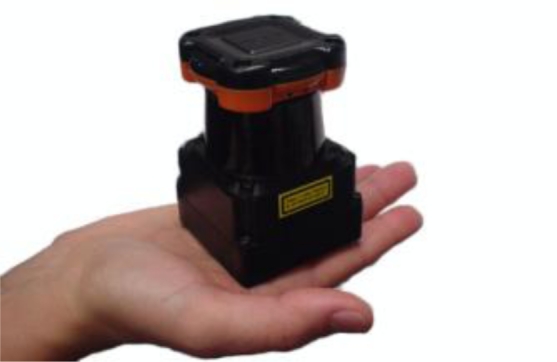
Hokuyo UTM-30LX.

A terrestrial LIDAR was used in this work to record the trajectory of the legs of the subjects. The selected LIDAR was the Hokuyo UTM-30LX [40] (Figure 1), a two-dimensional radial scanning laser range finder with an effective sensing range up to 30 meters in indoor and outdoor environments. The distance between the laser and the object is obtained measuring the elapsed time between the emission of a pulsed laser beam and the reception of its echo; this time is used to estimate the distance.

The UTM has a scanning range from −135° to +135° in steps of 0.25° (0° is in the front of the device) with a resolution of 1 mm, and one scan is completed in 0.025 s (40 Hz). The raw scan data provided by the device contains 1,081 distance-points expressed in millimeters and represented using 18 bits with a total of 3,243 bytes per scan.

The UTM was attached to an Intel® Core 2, 1.66 GHz, 1 GB RAM portable computer using a USB 2.0 port for registering data. The UTM was controlled using a proprietary (open) protocol based on messages. An interesting feature of the UTM is the inclusion of an internal reference time (1 ms resolution) in the raw data provided to avoid the effect of the delays originated in the USB communication with the computer.

**Figure 2.**
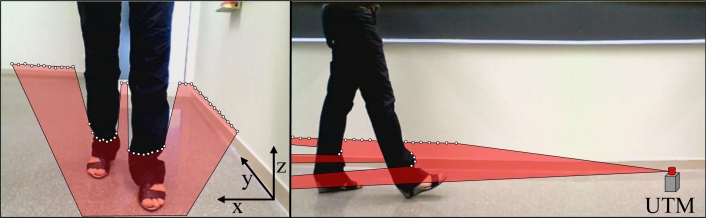
Representation of a typical measurement set with a representation of the scan plane.

**Figure 3.**
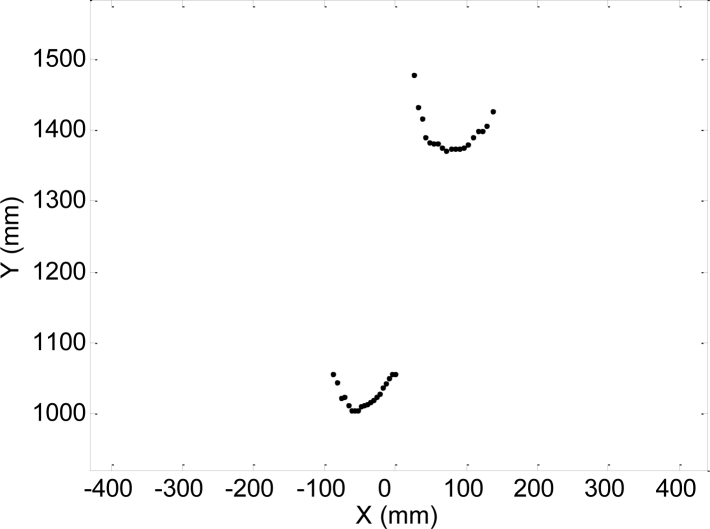
Example of LIDAR raw data points showing two legs.

The measurement procedure used to obtain the gait parameters from the scans acquired with the LIDAR is stated in [39]. Figure 2 shows a representation of a typical scanning procedure during a walking experiment. The LIDAR was placed at a height of 100 mm above the ground (approximately at ankle height) with the scanning plane parallel to the floor to obtain the maximum information from the legs without detecting the shoes/feet. Figure 3 shows an example of the raw data points obtained in the case of two isolated legs in front of the LIDAR. This raw data is segmented and used to fit two circles that define the instantaneous position of the legs of the subject while walking [39] and then the parameters of the gait are computed.

#### 2.2.2. Alcoholmeter

**Figure 4.**
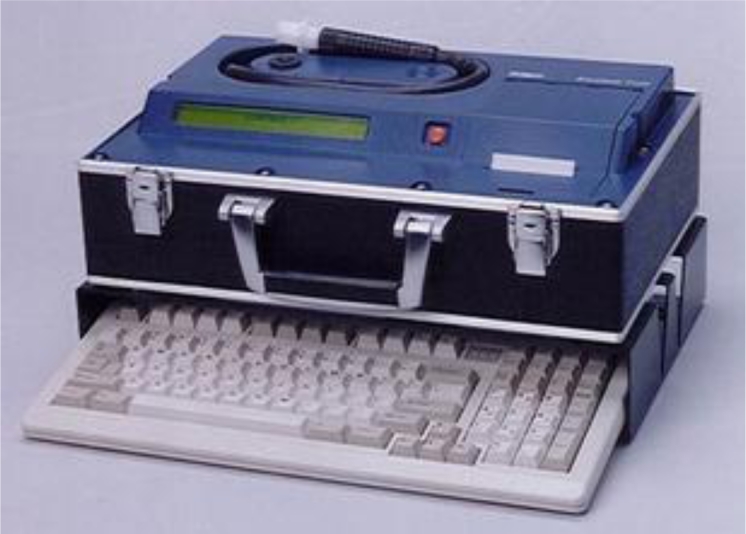
Alcotest 7110 Evidential.

An alcoholmeter was used in this work to correlate the measurement of the gait and trajectory parameters with the breath alcohol concentration. The selected alcoholmeter was the Alcotest 7110 Evidential (Figure 4) [35]. This alcoholmeter measures different parameters when the breathing air enters the instrument for analysis: the breath temperature, exhalation flow rate, the blowing volume and the breath-alcohol concentration. The blood alcohol concentration can be derived by applying a conversion factor (2,100) to the breath alcohol concentration. The Alcotest 7110 determines the breath-alcohol concentration using two different measuring systems: an infrared sensor and an electrochemical sensor. Both measuring systems use their respective sensors to independently measure the alcohol concentration in air exhaled from deep in the lungs, and as such, each system monitors the other. The measurement is only accepted if the results provided by both sensors correspond within very narrow limits.

According to the manufacturer, the Alcotest 7110 has a measurement range up to 3 mg/L (ethanol mass per breath volume at 34 °C and 1,013 hPa) with a resolution of 0.01 mg/L, and requires a breath volume of 1.5 L, a blowing time higher than 3 s, and 2 minutes to complete a measurement cycle.

The alcoholmeter was provided by the Lleida section of the Police Force of the Government of Catalonia (Mossos d’Esquadra). All breath alcohol intake measurements were performed by an experienced police officer that was also in charge of the equipment.

### 2.3. Experimental

Three specific experiments have been developed in this work. In each walking experiment, the volunteers were asked to walk 10 m in the LIDAR direction from a specific starting point; their walking trajectory was registered with the LIDAR for offline analysis.

In the first experiment, the volunteers were asked to maintain a straight, unsupervised and constant (same rhythm) walk in a corridor to provide reference gait parameters. In a second experiment, the volunteers were asked to maintain an unsupervised and constant walk, but following a path defined by a line painted into the floor. In this case the line has a sinusoidal shape with two periods: 6 and 12 m and amplitudes of 100, 200, 300 and 400 mm. The third experiment was proposed to evaluate a possible future application. Two selected volunteers (1 man and 1 woman) were asked to have perform a straight, unsupervised and constant walk in a corridor after some repetitive moderate alcohol intake. The main goal was to estimate their trajectory after some recent alcohol consumption. The subjects were asked to drink 40 mg of an alcoholic beverage (approximately 16 mg of alcohol), wait 5 minutes, perform the unsupervised walking experiment, measure breath alcohol concentration with the alcoholmeter, and then repeat the procedure after a new alcohol intake. The complete experiment lasted 30 minutes covering six complete walks and was developed following the safety recommendations of the police office in charge of the alcoholmeter.

### 2.4. Parameters

**Figure 5.**
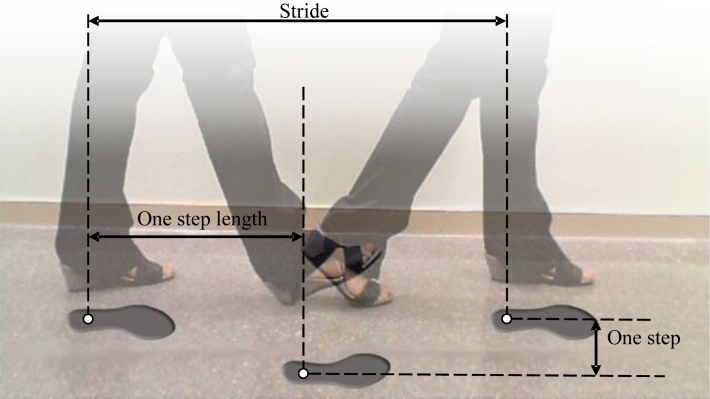
Gait parameters.

**Figure 6.**
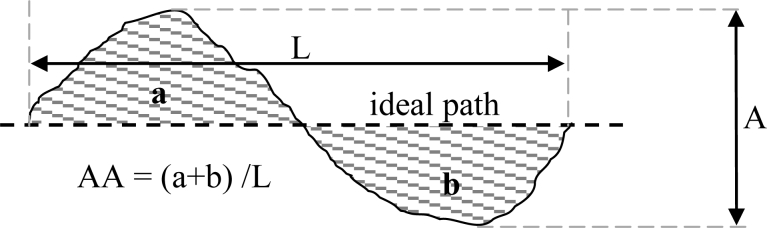
Graphic definition of the A and AA parameters.

Figure 5 shows a representation of the basic gait parameters analyzed in this work: stride (mm), the distance between successive points of initial contact of the same foot; one step width (mm), the width distance between the point of initial contact of one foot and the point of initial contact of the opposite foot; one step length (mm), the distance between the point of initial contact of one foot and the point of initial contact of the opposite foot; stride cycle (s), the time interval between successive points of initial contact of the same foot; left/right step length relationship (dimensionless), the coefficient of diving the left step length by the right one; stance phase time (s), the period of time between the point of initial contact of one foot with the floor and the point of final contact with the floor of the same foot; swing phase time (s), the period of time between the point of final contact of one foot with the floor and the point of initial contact with the floor of the same foot; stance/swing phase time relationship (dimensionless), the coefficient of diving the stance phase time by the swing one. Two specific trajectory parameters are also analyzed: the maximum amplitude (in mm) of the trajectory relative to a fitted straight path (labeled as A in Figure 6) and the normalized cumulative area (in mm) relative to a fitted straight path (labeled as AA in Figure 6). Alternatively, the alcoholmeter provided the instantaneous breath alcohol concentration (mg/L).

**Figure 7.**
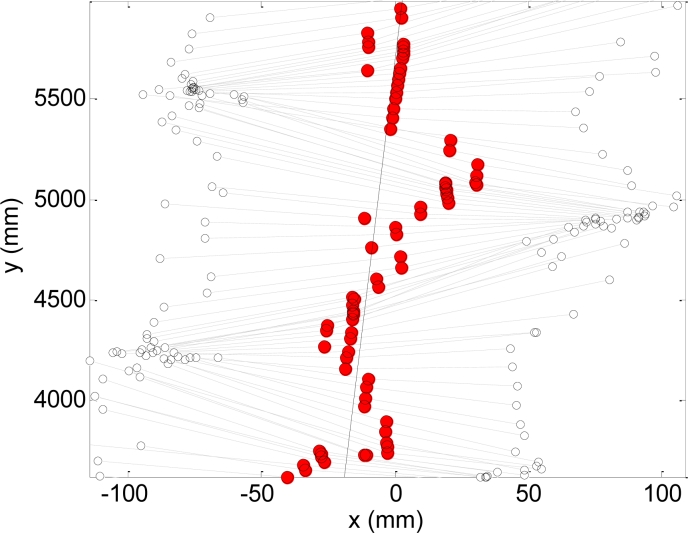
Graphic example of the procedure used to estimate the straight path.

Figure 7 shows a graphical representation of the data used to estimate the ideal straight path. The algorithm has two steps. In a first step the position of the center of both legs (white circles, joined by gray dotted lines) are averaged to get an estimate of the central position of the body (red circles). Then, these positions are applied to a least mean square algorithm to get the line (solid line) identified as the ideal straight path of the walking. The amplitude A and area AA are computed later relative to this ideal path (Figure 6).

## 3. Results and Discussion

Tables 1 to 6 show the results of the experiments performed in this work. The LIDAR provides information of several steps of the walk so the average and standard deviation of the main gait parameters are shown for each trajectory analyzed. This is not the case of the trajectory parameters A and AA that are computed only once per trajectory.

Tables 1 and 2 show the values of the gait parameters of the first experiment where the volunteers were asked to undertake a straight, unsupervised and constant walk. In both tables, the last row shows the average values obtained in the experiment. Table 1 shows the parameters obtained in four walks of three women and Table 2 shows the parameters of three men. In this experiment, the average value of the stride was 1,429 for women and 1,399 mm for men with an average standard deviation of 33.6 and 34.5 mm respectively. The results obtained in these unsupervised walking experiments show that each user has a typical average stride length but there were strong relative changes in the value of the standard deviation for the same volunteer, the maximum deviation was 46 for women and 54 mm for men. The average value of the step width was 175.5 mm and 173.7 mm for women and men with an average standard deviation of 25.6 and 19.6 mm respectively. The stride cycle had an average value of 1.03 and 1.04 seconds for women and men with no significant differences in the standard deviation. The relationship between the stance and the swing phase of the gait had average values of 1.74 for women and 1.76 for men. The average relationship between the left and right step length was 0.99 in both cases. Finally, the trajectory parameter A has average values of 143 mm for women and 140 mm for men whereas the cumulative parameter AA has average values of 22 and 25.6 mm respectively. In this work, these results can be considered small and reference values corresponding to an unsupervised straight walking trajectory.

**Figure 8.**
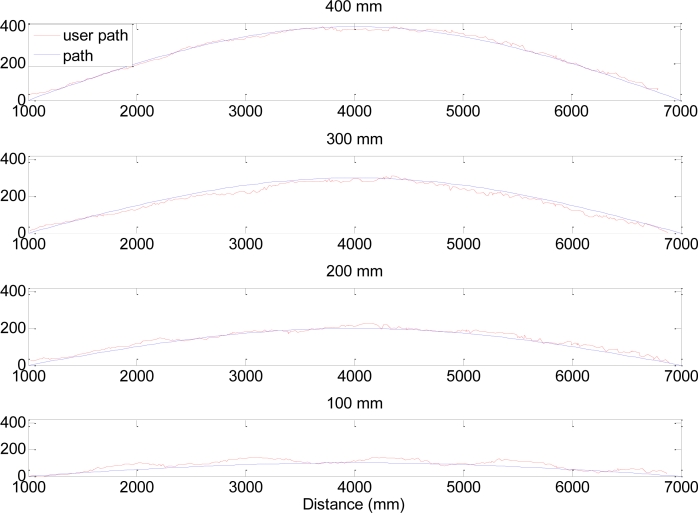
Example of trajectories registered: Half period case.

**Figure 9.**
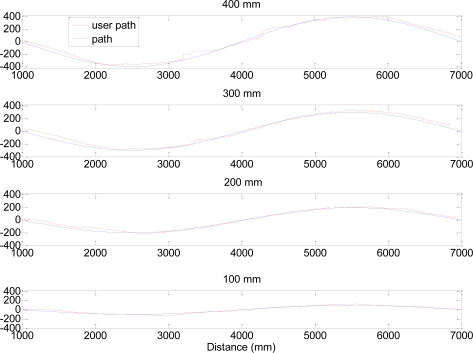
Example of trajectories registered: One period case.

Tables 3 and 4 show the average value of the gait parameters obtained in the second experiment. In this case 15 volunteers (8 male and 6 female) were asked to have carried out an unsupervised walk but following a line painted into the floor. Table 3 shows the results obtained when the line followed a half-period sinusoidal path (period = 12 m; Figure 8) and table 4 the results in the case of a complete sinusoidal period (6 m; Figure 9). In both tables, the column labeled “Amplitude” depicts the real sinusoidal amplitude of the line painted into the floor, and the last row shows the correlation between the amplitude of the oscillation and the average gait parameters obtained. The correlation coefficient is used as it measures the strength and the direction of a linear relationship between two sets of data. The correlation coefficient can be positive, if the two sets go up together, or negative, if one goes up while the other goes down. A correlation greater than 0.8 is generally described as strong, whereas a correlation less than 0.5 is generally described as weak.

**Figure 10.**
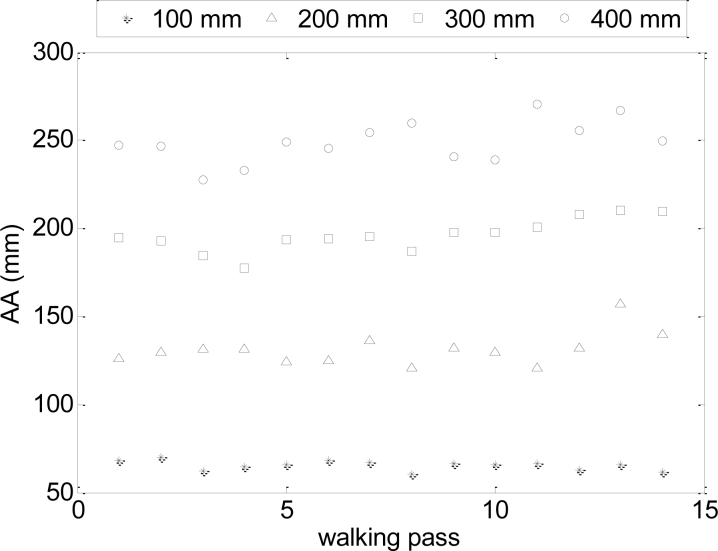
Individual values of the AA parameter: Half period case.

**Figure 11.**
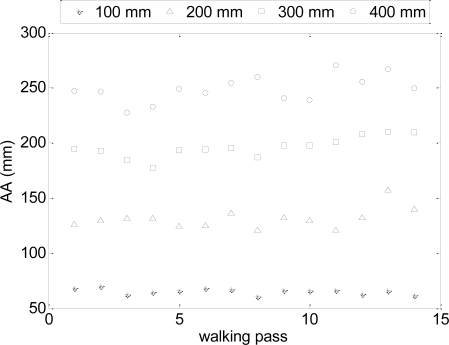
Individual values of the AA parameter: One period case.

Figure 8 and 9 show several trajectory examples with the proposed path (blue line) and the measured walking path (red line); the amplitude of the sinusoid is labeled in the title of the figures for reference. The standard deviations of the trajectories (proposed path *vs.* measured) shown were 30, 52, 16 and 11 mm for sinusoidal amplitudes of 400, 300, 200 and 100 mm respectively. Figures 10 and 11 summarize the values of the AA parameter obtained in each trajectory analyzed in Tables 3 and 4; the amplitude of the sinusoid is also labeled as a reference. The standard deviation of the trajectories (proposed path *vs.* measured) was 150, 91, 52 and 40 mm for the sinusoidal amplitudes of 400, 300, 200 and 100 mm, respectively.

There are very small divergences between the results shown in Tables 3 and 4 and the reference average values shown in Tables 1 and 2. In general, there is a weak or poor correlation between gait parameters and the amplitude of the sinusoidal of the path and, in the cases of strong correlation, it is not clear if this relationship is caused by the sinusoidal path or by the effect of fixing the attention in following a line painted into the floor. Alternatively, in both tables the average A and AA trajectory parameters have a strong correlation (>0.99) with the amplitude of the sinusoid. These results prove the utility of the proposed (A and AA) trajectory parameters to detect oscillating walking paths with a LIDAR.

Finally, Tables 5 and 6 show the results obtained in the third and last experiment. The gait and trajectory parameters were obtained for one woman and one man relative to moderate alcohol intake according to the procedure defined in Section 2.3. The last row of both tables shows the correlation between the measured breath alcohol concentration and the gait parameters obtained in each pass. The maximum breath alcohol concentration achieved during the experiments was 0.58 mg/L for the man and 0.91 mg/L for the woman. Currently, the legal breath alcohol limit for driving a car in our country (Spain) is 0.25 mg/L, so the concentrations reached in the experiments exceed this legal limit and can be representative in terms of a possible practical application of the proposed measurement system to preventively estimate if a subject is under the influence of some recent alcohol intake.

Results show that the standard deviation of the average stride length was very low (the walking cadence was very constant) with very weak correlation (0.25 for the woman and 0.46 for the man) with the breath alcohol concentration. Similar weak correlations were obtained for the step width, stride cycle, and step length ratio. In the case of a woman the standard deviation of the ratio between the stance and swing phase of the gait had a strong correlation (0.95) with the breath alcohol concentration but was nearly zero (0.05) in the case of a man.

**Figure 12.**
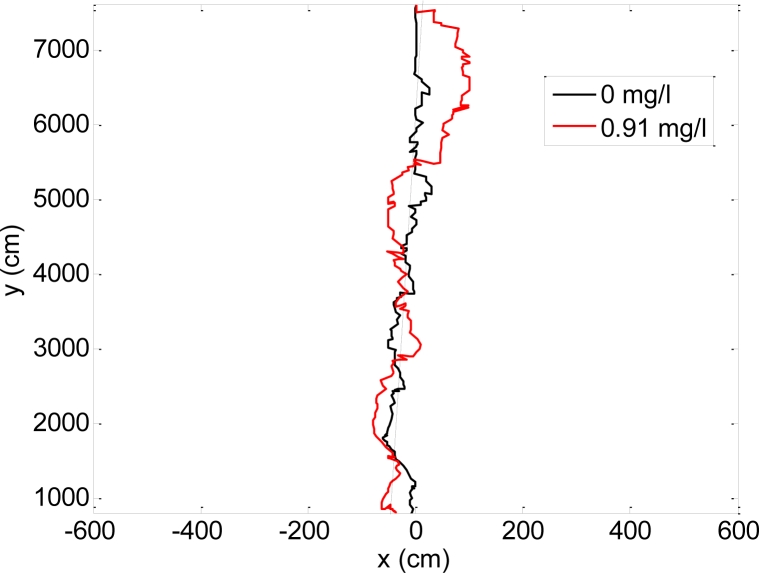
Details of two walking trajectories for different breath alcohol concentration: Woman case.

The results showed that the values of the parameters that define the amplitude of the oscillation, A and AA, were very small with a very weak correlation with the breath alcohol concentrations so all analyzed trajectories were very straight. These results coincide with the visual impressions obtained while observing the walking trajectory of the volunteers. These results are also in concordance with similar analyses performed with other measurement principles where very weak alteration of the walk was observed under the effect of moderate alcohol ingestion [32–34]. In this specific experiment, the duration (30 minutes) was probably too small to appreciate the depressant effect of the alcohol in the central nervous system and any resulting alteration in the gait and trajectory of the volunteers. Finally, Figure 12 shows a detail of two walking paths obtained with breath alcohol concentrations of 0 and 0.91 mg/L (woman case). In both cases, the walking paths were very similar despite the different breath alcohol concentrations measured with the alcoholmeter.

## 4. Conclusions and Future Work

This work proposes the use of a LIDAR laser range sensor in order to measure different unsupervised and oscillating walking paths. The gait and trajectory parameters were obtained with a terrestrial LIDAR placed 100 mm above the ground, with the scanning plane parallel to the floor. The estimate of the trajectory and gait parameters was obtained without attaching any markers to the subjects or modifying the floor in any way. The computation of two specific trajectory parameters is proposed to measure the oscillation of the walking trajectories: the maximum amplitude of the trajectory relative to a fitted straight path and the normalized cumulative area relative to a fitted straight path. Three different walking experiments were performed to test the proposed measurement system. In a first experiment, the unsupervised straight walk of some volunteers was measured, showing small deviations in the gait and trajectory parameters. In a second experiment, the unsupervised walk of some volunteers following oscillating paths painted into the floor was measured, obtaining small deviations in the gait parameters and estimating correctly the amplitude of the oscillation of the gait. In the third experiment, the unsupervised straight walk of two volunteers (one man and one woman) under the effect of moderate alcohol ingestion was measured as a preliminary proposal of a practical application of the measurement system. In this specific experiment the walking trajectories measured were very straight and these results were confirmed by the visual observations performed. These preliminary results showed that some gait parameters had a very strong correlation with the breath alcohol concentration. For example, the standard deviation of the ratio between the stance and swing phase of the gait had a strong correlation (0.95) in the case of a woman, but this correlation was very weak (0.05) in the case of a man. The standard deviation of the step width had a strong correlation (0.92) in the case of the man, but a weak and negative correlation (–0.33) in case of a woman. Therefore, this point requires further experimentation and analysis to extract general conclusions.

The main conclusion of this work is that a large and oscillating walking trajectory can be measured by using a terrestrial LIDAR and a minimum infrastructure without any previous contact with the subject. This conclusion can foster the development of a new range of non-intrusive and ambulatory applications based on the analysis of the walking path or gait parameters. As a future work, the complete kinematics of the gait will be analyzed and the measurement system will be compared with other alternative measurement systems.

## Figures and Tables

**Table 1.** Gait parameters of three women.

	**Stride**		**One step width**		**Stride cycle**		**Left/Right step length relationship**	**Stance/Swing phase time relationship**	**AA**		**A**	
**Women**	**Mean**	**Std**	**Mean**	**Std**	**Mean**	**Std**	**Mean**	**Mean**	**Mean**	**Std**	**Mean**	**Std**
User A	1581.33	31.18	172.01	37.04	1.04	0.010	1.00	1.60	25.228		122.48	
User A	1596.63	37.45	175.95	18.46	1.02	0.017	0.97	1.69	20.906	3.292	89.56	75.88
User A	1498.81	43.41	165.26	15.45	1.01	0.010	0.96	1.71	19.562		235.50	
User A	1559.82	33.13	141.6	23.82	1.01	0.002	1.00	1.64	17.430		235.24	
User B	1355.33	25.26	196.33	36.99	1.04	0.008	1.00	1.78	29.377		139.45	
User B	1388.71	44.87	175.58	20.13	1.08	0.014	0.94	1.81	33.583	10.92	120.72	33.00
User B	1364.79	34.69	163.61	29.06	1.11	0.026	0.95	1.83	35.645		140.80	
User B	1432.39	45.92	167.12	13.16	1.16	0.033	1.02	1.76	11.650		70.24	
User C	1318.77	37.68	199.97	36.10	0.98	0.029	0.99	1.75	16.342		85.45	
User C	1328.88	16.67	176.06	16.08	0.98	0.011	1.04	1.80	21.488	2.56	100.96	69.08
User C	1335.68	19.40	179.27	25.61	0.96	0.011	1.00	1.74	16.129		137.17	
User C	1394.50	33.38	193.15	35.88	0.95	0.009	0.97	1.73	16.696		239.05	
Average	1429.6	33.59	175.5	25.65	1.03	0.015	0.99	1.74	22	5.59	143.05	59.32

**Table 2.** Gait parameters of three men.

	**Stride**		**One step width**		**Stride cycle**		**Left/Right step length relationship**	**Stance/Swing phase time relationship**	**AA**		**A**	
**Men**	**Mean**	**Std**	**Mean**	**Std**	**Mean**	**Std**	**Mean**	**Mean**	**Mean**	**Std**	**Mean**	**Std**
User D	1289.10	39.52	223.04	18.16	1.05	0.021	0.99	1.75	27.50		121.78	
User D	1241.80	21.67	218.41	16.66	1.08	0.033	1.00	1.72	28.45	8.12	150.75	75.88
User D	1295.78	39.48	196.44	32.13	1.02	0.022	0.95	1.75	44.63		220.00	
User D	1277.30	25.28	171.06	28.82	1.03	0.018	0.91	1.84	37.66		151.25	
User E	1539.55	20.13	192.78	12.18	1.09	0.005	0.99	1.56	23.196		109.609	
User E	1565.08	27.08	168.87	11.98	1.06	0.007	0.99	1.65	27.913	5.55	178.403	33.00
User E	1600.47	54.17	155.57	24.97	1.07	0.010	0.99	1.59	16.813		89.129	
User E	1578.08	21.26	127.45	18.93	1.06	0.001	1.00	1.67	29.016		135.276	
User F	1316.02	44.95	159.62	12.06	1.00	0.018	1.03	1.85	30.547		224.354	
User F	1355.49	32.60	162.05	23.00	1.01	0.013	1.03	1.96	17.406	8.62	100.368	69.08
User F	1359.73	48.75	162.00	20.23	1.00	0.012	1.01	1.88	12.226		85.581	
User F	1376.58	39.00	147.65	15.76	1.00	0.013	0.99	1.89	12.315		115.458	
Average	1399.6	34.49	173.75	19.57	1.04	0.014	0.99	1.76	25.63	7.43	140.16	47.77

**Table 3.** Gait parameters obtained following a sinusoidal path (half period).

**Amplitude**	**Stride**		**One step width**		**Stride cycle**		**Left/Right step length relationship**	**Stance/Swing phase time relationship**	**AA**		**A**	
	**Mean**	**Std**	**Mean**	**Std**	**Mean**	**Std**	**Mean**	**Mean**	**Mean**	**Std**	**Mean**	**Std**
100 mm	1479.8	202.51	134.02	23.06	1.13	0.093	1.45	1.81	77.90	6.20	143.09	15.57
200 mm	1497	180.76	147.42	24.50	1.14	0.098	1.20	1.83	142.93	10.96	234.24	17.94
300 mm	1508	212.63	138.21	26.45	1.15	0.083	1.55	1.75	179.67	10.40	310.78	17.55
400 mm	1437.3	208.67	144.39	25.03	1.12	0.090	1.52	1.91	259.10	8.39	403.65	14.03
Correlation	−0.484	0.457	0.468	0.724	−0.372	−0.494	0.461	0.427	0.991	0.359	0.999	−0.353

**Table 4.** Gait parameters obtained following a sinusoidal path (one period).

	**Stride**		**One step width**		**Stride cycle**		**Left/Right step length relationship**	**Stance/Swing phase time relationship**	**AA**		**A**	
**Amplitude**	**Mean**	**Std**	**Mean**	**Std**	**Mean**	**Std**	**Mean**	**Mean**	**Mean**	**Std**	**Mean**	**Std**
100 mm	1467.6	248.67	150.33	30.17	1.13	0.114	1.05	1.83	66.24	5.59	268.08	23.16
200 mm	1531.1	271.72	172.66	36.16	1.12	0.145	1.64	1.96	130.50	8.77	461.99	32.10
300 mm	1524.8	179.14	162.73	20.49	1.19	0.153	1.60	1.90	196.89	10.1	663.65	40.99
400 mm	1464.9	259.74	167.43	22.67	1.18	0.138	1.38	1.94	249.68	14.9	800.88	34.92
Correlation	−0.052	−0.184	0.559	−0.686	0.822	0.613	0.448	0.658	0.998	0.97	0.996	0.768

**Table 5.** Gait parameters relative to alcohol intake: Woman case.

**Woman**	**Stride**		**One step width**		**Stride cycle**		**Left/Right step length relationship**	**Stance/Swing phase time relationship**	**AA**	**A**
	**Mean**	**Std**	**Mean**	**Std**	**Mean**	**Std**	**Mean**	**Mean**	**Value**	**Value**
0	1514.45	14.76	193.40	27.26	1.13	0.007	0.98	1.61	24.42	112.80
0.13	1540.09	58.37	204.65	32.33	1.14	0.012	0.94	1.65	18.33	123.57
0.24	1533.86	26.03	216.04	20.74	1.11	0.011	1.04	1.72	15.17	140.46
0.39	1569.45	22.10	214.27	21.13	1.09	0.017	1.02	1.74	26.81	134.40
0.51	1507.10	63.19	147.40	19.77	1.18	0.008	1.03	1.71	16.07	90.75
0.68	1554.62	38.22	126.16	20.59	1.16	0.013	1.02	1.81	21.52	135.40
0.91	1567.97	38.33	165.55	26.73	1.13	0.018	1.02	1.86	21.19	139.96
Correlation	0.35	0.25	−0.76	−0.33	0.48	0.62	0.53	0.95	0.00	0.27

**Table 6.** Gait parameters relative to alcohol intake: Man case.

**Man**	**Stride**		**One step width**		**Stride cycle**		**Left/Right step length relationship**	**Stance/Swing phase time relationship**	**AA**	**A**
	**Mean**	**Std**	**Mean**	**Std**	**Mean**	**Std**	**Mean**	**Mean**	**Value**	**Value**
0	1337.93	25.56	164.12	27.41	1.01	0.009	1.00	1.93	70.67	308.91
0.06	1396.53	27.49	173.15	33.27	0.93	0.007	1.00	1.89	100.31	444.03
0.07	1395.50	30.46	121.70	17.95	0.96	0.017	0.98	1.86	24.37	166.28
0.13	1415.49	55.31	152.05	31.09	0.96	0.008	0.91	1.99	49.61	245.30
0.37	1455.06	26.79	122.15	42.51	0.98	0.010	0.89	1.92	52.36	228.44
0.40	1459.19	38.94	157.14	44.98	1.00	0.011	0.96	1.94	52.78	271.55
0.58	1353.91	52.36	150.31	62.80	1.06	0.006	1.01	1.90	72.90	315.40
Correlation	0.25	0.46	−0.20	0.92	0.70	−0.29	−0.06	0.05	−0.01	−0.07
